# Female reproductive fluid and male seminal fluid: a non-gametic conflict for post-mating control

**DOI:** 10.1098/rsbl.2023.0306

**Published:** 2023-09-27

**Authors:** Livia Pinzoni, Lisa Locatello, Clelia Gasparini, Maria Berica Rasotto

**Affiliations:** ^1^ Department of Biology, University of Padova, Padova 35131, Italy; ^2^ Department of Biology and Evolution of Marine Organisms (BEOM), Stazione Zoologica Anton Dohrn, Fano Marine Center, Fano 61032, Italy

**Keywords:** non-gametic components, reproductive fluids, sexual selection, sperm competition, grass goby

## Abstract

Growing evidence shows that non-gametic components released by both males and females can significantly drive sperm competition outcomes. Seminal fluid (SF) was shown to influence paternity success by affecting rival males' sperm performance, and, in some species with male alternative reproductive tactics, to selectively decrease the fertilization success of males of the opposite tactic. Female reproductive fluid (FRF) has been proven to differentially influence ejaculates of different males and bias fertilization towards specific partners. Whether, and with what outcome, these two processes can intersect to influence sperm competition is still unknown. Here we explore this scenario in the grass goby (*Zosterisessor ophiocephalus),* a fish with territorial–sneaker reproductive tactics, where sneaker males can exploit the territorials’ SF while penalizing territorial sperm performance with their own fluid. To test whether FRF can rebalance the ejaculate competition in favour of territorial males, we used *in vitro* fertilization with a SF mixture (territorial + sneaker), using increasing concentrations of FRF, to simulate the natural conditions that ejaculates encounter towards the eggs. Our findings revealed a differential effect of FRF on the different tactics' fertilization success, favouring territorial ejaculates, possibly through an attenuation of the detrimental effects of sneaker SF, and enabling females to regain control over the fertilization process.

## 1. Introduction

Sperm competition, occurring whenever the ejaculates of rival males compete to fertilize the same eggs [1], is a pervasive evolutionary force able to shape male behaviour, physiology, morphology, and to influence ejaculate production and allocation strategies [2–7]. Recently, studies on sperm competition have begun exploring the role of non-gametic components, starting from male seminal fluid (SF), which can influence sperm competition by affecting female receptivity, oviposition rate, and remating possibilities, as well as the performance of rival sperm [8–10]. SF was indeed shown to either equally improve own and rival sperm viability [11–13], or specifically incapacitate the sperm of rival males in insects [14,15]. In two fish species with male alternative reproductive tactics (ARTs), the grass goby (*Zosterisessor ophiocephalus*) and the Chinook salmon (*Oncorhynchus tshawytscha*), a tactic-specific detrimental effect of SF on rival sperm was also observed: sneaker males can exploit the territorials’ SF and, in the case of the grass goby, even penalize territorial sperm performance with their own fluid [16,17].

This SF effect opposes female pre-mating preferences for territorial males, potentially setting the stage for sexual conflict and for coevolutionary processes in which female post-mating mechanisms are expected to re-balance the competition in favour of initially preferred males.

Sperm competition processes always take place in a reproductive environment shaped by the females, even in external fertilizers, where fertilization is nonetheless influenced by the female reproductive fluid (FRF) released with the eggs. FRF is the medium, surrounding the eggs before and during fertilization, and encountered by sperm on their way towards the eggs [18]. This fluid has been shown to differentially influence sperm competitiveness of different males, thereby mediating cryptic female choice, across a variety of different species [19–23]. In fish, FRF can bias fertilization towards ejaculates of unrelated [24], or genetically compatible males [25], and even of preferred male phenotypes in a fish species with ARTs [26].

Despite growing interest in the role of FRF in post-mating sexual selection, implications of the interplay between FRF and male SF during fertilization remain unexplored.

Here, we use the grass goby *Z. ophiocephalus* to explore the potential of FRF to interfere with the dynamic of tactic-specific sperm competition impairment mediated by SF. This species exhibits external fertilization and male ARTs, with territorial males (preferred by females at the pre-mating level) building and defending a nest, courting females, and providing parental care to the eggs, and sneaker males parasitizing the territorial spawnings to steal some fertilizations [27,28]. Territorial males release sperm in the form of mucous trails on the nest ceiling, where females lay their eggs one at a time. Conversely, sneaker males usually release their fluid ejaculates from an unfavourable position, further away from the spawning female. To compensate for this positional disadvantage, and for always having to mate under competition [29] sneaker males invest disproportionately more in sperm production than territorials, that in turn produce more SF [30]. As a result, territorials' ejaculates slowly dilute in seawater, ensuring a constant supply of active sperm throughout the egg deposition process, and enabling them to focus on nest defence [31].

A recent study revealed a concentration-dependent effect of FRF on both sneaker and territorial sperm performance, with sperm released near the eggs, thus encountering higher FRF concentrations, significantly increasing their velocity and motility [32]. The ability to release sperm close to the eggs is usually the prerogative of territorial males that would thus be favoured at the post-mating level by FRF. However, sneaker males reaching the proximity of territorial males gain a higher fertilization success by enjoying an increase in sperm performance exploiting the territorials' SF, and, in turn, decreasing, with their fluid, the territorials’ sperm fertilizing ability [16].

To understand whether, in the grass goby, FRF could re-balance the ejaculates competition and attenuate the impairment of territorials' sperm performance by sneaker SF, we designed an *in vitro* fertilization experiment simulating the condition of natural competition that ejaculates encounter in their journey towards the eggs. By *in vitro* fertilizations (IVFs) we tested the fertilizing ability of sperm of sneaker and territorial males exposed to two different concentrations of FRF and in the presence of both own and rival males' SF (details on the design rationale can be found in the electronic supplementary material file [33]).

## 2. Material and methods

Grass goby females, territorial and sneaker males were sampled inside nests, in the Venetian Lagoon, during their breeding season (March–May 2022). In the field, males were initially categorized as territorials or sneakers following [32]. All individuals were then transferred to the Hydrobiological Station ‘Umberto D'Ancona’ (Chioggia, Italy), and kept, for 5 days max., in separate tanks with continuous water exchange (20 ± 1°C) under a 14 L : 10 D photoperiod and fed twice/day with 2 or 3 (according to body size) fresh mussels each.

The day of the experiment, all individuals were anaesthetized using MS 222 (tricaine sulfate; Sandoz, specific dosage varying with body size following [34]), weighed and measured. Gametes were collected following [16]. Briefly, eggs were obtained through a gentle pressure on the swollen abdomen of ready-to-spawn females and collected, surrounded by FRF, on acetate sheets of standard length. Ejaculates were obtained by gently pressing on the abdomen of males, collected with a Gilson pipette, and centrifuged at 13 300*g* for 3 min at 4°C to separate SF from the sperm cells that were then re-suspended in an extender inactivating medium [35]. The field attribution of males' tactics was validated for all males according to their ejaculates’ characteristics (fluid and white in sneakers, because of the high sperm content, dense and opaque in territorials, due to the lower sperm count and higher mucin content) and sperm production (sperm number of sneakers = 1.56 × 10^6^ ± 1.08 × 10^5^, territorials = 5.34 × 10^5^ ± 6.19 × 10^4^; assessed with a LUNA Automated Cell Counter - Logos Biosystems).

**Figure 1.**
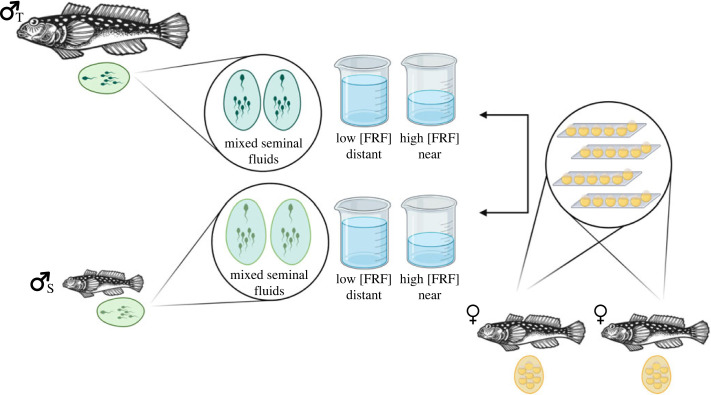
Overview of experimental design. Fertilization success of sneaker and territorial males was compared in the presence of a mixture of both SF (simulating competition) across two treatments: high and low concentration of FRF (simulating, respectively, proximity or distance from the eggs). For each replicate (consisting of one sneaker, one territorial, and two females) we collected the eggs (standardized number) of each female on four acetate sheets (total eight sheets) that were randomly assigned to the different treatments. Then, using two glass beakers containing filtered marine seawater (with one volume twice as large as the other), we performed individual IVFs as described above. Fertilization success of each male across treatments was then individually recorded. Total number of replicates = 17. Made with Biorender.

For each male, subsamples of 10 µl of sperm solution were activated with 20 µl of filtered seawater and incubated for 2 min with 2 µl of SF (1 µl own + 1 µl of the rival of the opposite tactic). Sneakers' SF was pre-diluted 10-fold in filtered seawater before use to match the natural SF concentration of sneaker and territorial males [16]. A volume of sperm solution containing 8 × 10^5^ sperm cells was then taken, diluted to 50 µl with filtered seawater, and used for IVFs. These were performed by placing two acetate sheets, carrying the eggs of two different females (to minimize the potential male-by-female interaction effects [36]), on the bottom of a glass beaker containing 500 or 250 ml of filtered seawater, simulating the two different scenarios of sperm being respectively distant or close from the eggs (figure 1) (i.e. experiencing different FRF concentrations). Sperm were homogeneously deposited on the water surface with a Gilson pipette and left to fertilize the eggs for 15 min. Afterwards, the sheets were extracted, gently washed and placed in a new glass beaker with clean filtered seawater and oxygen supply. Fertilization success was checked at 4 h post fertilization, when the complete lifting of chorion and the first stages of cellular division are clearly visible. For each IVF, 259.04 ± 2.25 eggs were used. Afterwards, all individuals were released at the site of collection unharmed.

Statistical analyses were conducted using R v 3.6.3 [37]. The proportion of fertilized eggs across treatments was analysed using a linear mixed effect model (‘lmer’ function of the ‘lme4’ package [38]), with treatment (‘high’ and ‘low’ concentrations of FRF) and male reproductive tactic as fixed factors, and male and female identity as random factors with fixed intercept. The associated *p-*values of the fixed factors were obtained from the ‘anova’ function of the ‘lmerTest’ package [39] using Satterthwaite's approximation to calculate the denominator degrees of freedom. *Post-hoc* pairwise comparisons were performed with the function lsmeans (package ‘lsmeans’ [40]), applying a Tukey correction for multiple comparisons. The inspection of residuals' distribution using the package ‘DHARMa’ [41] indicated that the linear model met the assumptions. All means are shown with associated standard error.

## 3. Results

We found a significant effect of increasing concentrations of FRF (*F*_15.798_ = 17.97, *p* < 0.001), of male mating tactic (*F*_31.531_ = 31.53, *p* = 0.002) and, notably, of their interaction (*F*_16.254_ = 48.33, *p* < 0.001) on fertilization success (in presence of a mixture of own SF and of SF of the opposite tactic). This indicates that males' fertilization success was differentially affected by the increasing of FRF concentration according to the mating tactic adopted (territorial or sneaker).

**Figure 2.**
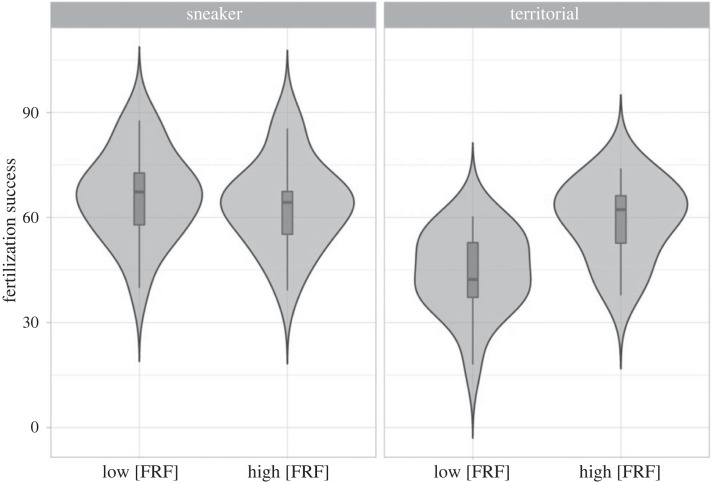
Effect of increasing concentrations of FRF ([FRF]) on the fertilization success of territorial and sneaker males. Violin plots show the distribution of the variable ‘fertilization success’, box plots show the median, interquartile range, and upper and lower extremes of the variable. *N* = 17 territorials and 17 sneakers.

Specifically, at lower FRF concentration (i.e. simulating a greater distance from the eggs) sneaker males were significantly more successful in fertilization than territorials. This difference between tactics disappeared in the presence of higher concentrations of FRF (i.e. simulating higher proximity to the eggs) thanks to the significant increase of territorial males' fertilization success (table 1, figure 2) from 44.55 ± 2.74% to 56.84 ± 3.47%, whereas sneakers' fertilization success slightly decreased, although not significantly, from 66.17 ± 3.02% to 63.49 ± 2.99% (table 1, figure 2).

**Table 1.** *Post-hoc* pairwise comparisons of the effect of FRF increasing concentrations ([FRF]) on the fertilization success of sneaker and territorial males across all treatment × tactic combinations. Significant effects (i.e. cases where *p* < 0.05 and the 95% CI do not overlap zero) are highlighted in bold.

contrasts	estimate	s.e.	*t*	*p*	95% CI
sneaker low [FRF]– sneaker high [FRF]	0.027	0.016	1.717	0.332	−0.016 to 0.069
sneaker low [FRF]– territorial low [FRF]	0.216	0.043	5.006	**<0**.**001**	**0.100 to 0.333**
sneaker low [FRF]– territorial high [FRF]	0.093	0.043	2.151	0.157	−0.024 to 0.210
sneaker high [FRF]– territorial low [FRF]	0.189	0.043	4.369	**<0**.**001**	**0.073 to 0.306**
sneaker high [FRF]– territorial high [FRF]	0.066	0.043	1.536	0.428	−0.050 to 0.183
territorial low [FRF]– territorial high [FRF]	−0.123	0.016	−7.862	**<0**.**001**	−**0.165 to** −**0.081**

## 4. Discussion

Previous studies on grass goby fertilization dynamics highlighted the influence of either SF or FRF on the sperm performance of both territorial and sneaker males [16,32]. The cross interaction of rival males' SF was shown to differentially affect sperm performance, in terms of velocity and fertilization success, according to the tactic adopted by males [16]. The performance of territorial males' sperm was indeed shown to be negatively affected by the SF of sneaker males, while sneaker sperm were shown to exploit territorial male SF, overall displaying a higher fertilization rate. In turn, FRF alone always enhances the sperm performance of territorial males, regardless of its concentration, while sneakers' sperm enjoy increased performance only in presence of a higher FRF concentration, i.e. when in close proximity to the female [32]. Here, our contribution brings us a step closer to solving the puzzle of the interaction between male and female reproductive fluids in the game of competition. We evaluated the effect, on male fertilization success, of the simultaneous presence of rival male SF and FRF, the latter supplied at two different concentrations simulating those hypothetically encountered by sperm when released closer or at a greater distance to the eggs.

The results show that in presence of rival SF but at low FRF concentration, sneaker males have a higher fertilization success than territorials, whereas at higher FRF concentration the territorials enjoy an increase in fertilization success, reaching values similar to those of sneakers. Therefore, when the FRF level is low, sperm fertilizing ability (and thus, potentially, the outcome of male competition) appears to be primarily influenced by the effect of the interaction between rival males' SFs [16], and overall sneakers outclass territorial males. By contrast, when FRF concentrations increase (an inevitable occurrence during the fertilization process, when sperm swim progressively closer to the eggs) the detrimental effect of sneaker SF on territorial sperm is seemingly reduced, so that the fertilization success of territorial males increases, compared to that at low FRF concentration, and catches up with that of sneaker males. This suggests a FRF compensatory effect, attenuating the influence of sneaker SF on territorial sperm. Females, through their reproductive fluid, appear to be able to rebalance the ejaculates' competition and ultimately sustain their pre-mating preference for territorial males.

The observed effect is likely mediated by molecular interactions between the female and male fluids, which will certainly need further investigation. Little is known so far about the mechanisms underlying FRF-mediated paternity biases, even though some FRF-specific proteins, (e.g. glycoproteins), have been identified [42–44], indicating the potential for a specific function in the FRF interaction with ejaculates [18]. Specific protein components of FRF that could be responsible for the interaction with SF have been proposed by studies of proteomic analysis and comparison of evolution rates [45–48], but only in relation to the female reproductive tract in internal fertilizers (e.g. the mammalian oviductal glycoproteins [49]).

Not much is known about the components of sneaker SF impairing territorial sperm competitiveness, with which FRF could potentially interact to compensate for this effect. Only a few studies have investigated the composition of SF in relation to sperm competition in fish, suggesting that proteins with a molecular weight less than 50 kDa, as well as monosaccharides and triglycerides, could affect sperm performance [50,51].

Furthermore, the ionic composition of both SF [52] and FRF [53,54] has been proven to have important effects on sperm performance, even though, as of now, nothing is known about the consequences of these interacting fluids on the ionic composition of the ultimate fertilization medium.

Regardless of the specific mechanisms, our results highlight the importance of integrating both male and female evolutionary perspectives of ejaculate competition, as well as non-gametic components, that are emerging as pivotal players of post-mating sexual selection and sexual conflict.

## Data Availability

Raw data are available from the Dryad Digital Repository: https://dx.doi.org/10.5061/dryad.0p2ngf26d [55]. Details on the design rationale can be found in the electronic supplementary material file [33].
